# Immortalization of Fetal Bovine Colon Epithelial Cells by Expression of Human Cyclin D1, Mutant Cyclin Dependent Kinase 4, and Telomerase Reverse Transcriptase: An *In Vitro* Model for Bacterial Infection

**DOI:** 10.1371/journal.pone.0143473

**Published:** 2015-12-01

**Authors:** Kengo Kuroda, Tohru Kiyono, Emiko Isogai, Mizuki Masuda, Moe Narita, Katsuya Okuno, Yukako Koyanagi, Tomokazu Fukuda

**Affiliations:** 1 Graduate School of Agricultural Science, Tohoku University, 1–1 Tsutsumidori-Amamiyamachi, Aoba-ku, Sendai, Miyagi, Japan; 2 Division of Carcinogenesis and Cancer Prevention, National Cancer Center Research Institute, 5-1-1 Tsukiji, Chuo-ku, Tokyo, Japan; University of Newcastle, UNITED KINGDOM

## Abstract

Cattle are the economically important animals in human society. They are essential for the production of livestock products such as milk and meats. The production efficiency of livestock products is negatively impacted by infection with zoonotic pathogens. To prevent and control infectious diseases, it is important to understand the interaction between cattle tissue and pathogenic bacteria. In this study, we established an in vitro infection model of an immortalized bovine colon-derived epithelial cell line by transducing the cells with lentiviral vectors containing genes encoding cell cycle regulators cyclin D1, mutant cyclin dependent kinase 4 (CDK4), and human telomerase reverse transcriptase (TERT). The established cell line showed continuous cell proliferation, expression of epithelial markers, and an intact karyotype, indicating that the cells maintained their original nature as colon-derived epithelium. Furthermore, we exposed the established cell line to two strains of *Salmonella enterica* and EHEC. Interestingly, *S*. Typhimurium showed higher affinity for the established cell line and invaded the cytoplasm than *S*. Enteritidis. Quantitative RT-PCR revealed that gene expression of Toll-like receptor 1 (TLR1), TLR 2 and TLR 3, whereas TLR 4, 5 and 6 were not detectable in established cells. Our established immortalized colon-derived epithelial cell should be a useful tool for studies evaluating the molecular mechanisms underlying bacterial infection.

## Introduction

Cattle are the economically important domestic animal source of livestock-related products such as meat and milk. The efficiency of their production is largely affected by various zoonotic pathogens such as *Escherichia coli* O157:H7 [1] and *Salmonella enterica* [2]. Infection can be established by a variety of routes, including fecal contamination of feed, or transmission from humans or wild animals. Many pathogens in cattle are living as commensal bacteria at the mucosal surface without invading the reservoir host, however, exponential growth of the bacteria and invasion into the intestinal epithelial cells are critical steps to establish infection in infected animals. An in vitro cell culture system is essential for molecular studies of bacterial affinity for epithelial cells. However, as far as we know, intestinal cell lines from cattle are not available from worldwide cell banks such as the American Type Culture Collection (ATCC). Bacterial adhesion and invasion are detectable using relatively simple methods such as fluorescent immunohistochemical staining [3]. Thus, an established bovine colon epithelial cell line would be a powerful tool for studies that assess the effects of infectious bacteria on host colon epithelial cells.

Over the past several decades, primary cells have typically been immortalized by the introduction of Simian Vacuolating Virus 40 large T antigen, or human telomerase reverse transcriptase (TERT) with human papilloma virus-derived E6/E7 protein [4–6]. Although expression of these oncogenic proteins is effective for immortalization, these oncogenic proteins promote genomic instabilities such as chromosome structure abnormalities [7, 8]. Furthermore, the expression of these oncogenic proteins can change the original nature of the primary cells. Recently, Sasaki et al. and Shiomi et al. have demonstrated that co-expression of the human cyclin D1, mutant CDK4 (CDK4R24C), and TERT immortalizes human ovarian epithelial cells and myogenic cells [9, 10]. We also previously demonstrated that co-expression of human cyclin D1, mutant CDK4, and TERT efficiently immortalizes fibroblast cells derived from several kinds of animals such as pigs, cattle [11], and monkeys [12]. This immortalization was effective, regardless of the cell or tissue type or the species of origin, and retained the original karyotype pattern in a high percentage of the cells. Thus, this method is an excellent system for establishing cell lines that keep their original phenotypes.

## Materials and Methods

### Ethics

This study is one of the national projects associated with the Great East Japan Earthquake and has been entirely endorsed and supported by the Japanese government through the Ministry of Education, Culture, Sports, Science and Technology, Japan, and the detailed description of the animal care and protocols is described in our previous study [13]. In briefly, we collected organs and tissues from the euthanized cattle by the combined unit of veterinary doctors belonging to the Livestock Hygiene Service Center (LHSC) of Fukushima Prefecture and those belonging to the Ministry of Agriculture, Forestry and Fisheries, Japan. Cattle were sacrificed by these veterinarians by the following method according to the Regulation for Animal Experiments and Related Activities at Tohoku University (Regulation No: 2014kado-037). Cattle were sacrificed by exanguination from the jugular vein in their unconscious state by a pentobarbital (2 mg/kg) and suxamethonium (10 ml/kg) after intramuscular injection of hypnotics (xylazine hydrochloride, 0.2 mg/kg).

### Isolation of fetal bovine colon cells and primary culture

Colon epithelial tissues were obtained from a fetus of Japanese Black cattle (male, about 5 age in month), which was resected from euthanized parent cattle that were raised in the evacuation zone surrounding the Fukushima Daiichi Nuclear power plant accident. All procedures were authorized by the Animal Experiments and Related Activities Office at Tohoku University (Regulation No: 2014kado-037). The colon tissue was cut in parallel to intestinal tract that is 3 cm long in inside 1cm from anus. The tissue was gently washed with phosphate buffered saline (PBS) (NISSUI PHAMACEUTICAL CO., LTD., Tokyo, Japan). The epithelial layer including mucosa was scraped with a sterilized knife into a 100 mm dish coated with atelocollagen (KOKEN CO., LTD, Tokyo, Japan) and containing Dulbecco’s modified Eagle’s medium (Nacalai Tesque, Kyoto, Japan) supplemented with 10% fetal bovine serum (Invitrogen, Carlsbad, US) and 1% Antibiotic-Antimycotic Mixed Solution (Nacalai Tesque). The dish maintained at 37°C in an atmosphere containing 5% CO_2_ and medium change was conducted every three days.

### Viral vector construction and genetic transduction

To immortalize fetal bovine colon cells, CSII-CMV-TERT, CSII-CMV-cyclin D1, and CSII-CMV-hCDK4R24C were simultaneously introduced into the primary cells. To monitor the transfer efficiency, CSII-CMV-EGFP was introduced into the primary cells in the independent well. The preparation and recombination of lentiviral constructs have previously been described [9]. The production of recombinant lentiviruses with vesicular stomatitis virus G glycoprotein was also described in a previous study [9]. Primary cells were seeded at a density of 1.0×10^5^ cells/well in a 6-well plate and inoculated with CSII-CMV-TERT, CSII-CMV-cyclin D1, and CSII-CMV-CDK4R24C lentiviruses at a multiplicity of infection (MOI) of five for each virus in the presence of 6 μg/mL polybrene.

### Population doubling assay

Primary bovine colon cells and newly established bovine fetal colon epithelial cells in this study (BFCE-K4DT cells) were seeded at a density of 1×10^5^ cells/well on a 6-well plate (BD Biosciences, Franklin Lakes, US). When the cells reached confluency, both primary cells and recombinant cells were harvested, and the total number of cells in each well was determined using a Coulter automated cell counter (Invitrogen). Population doubling (PD) was used as the measure of cell growth rate. PD was calculated from the formula PD = log2 (A/B), where A is the number of harvested cells and B is the number of plated cells [6, 14]. Experiments were carried out in triplicate, and the averages and standard deviations (SD) were calculated.

### Cell cloning

After population doubling assay, BFCE-K4DT cells were seeded at a density of 100 cells to 100 mm dish, and cultured for 1 week. Cloning of BFCE K4DT cells from single cell were performed by cloning cylinders methods. Cells were washed with PBS. The cloning cylinder (SIGMA, St. Louis, US) was set gently over a colony and 100 μL 0.25% trypsin (Nacalai Tesque) was added to the cloning cylinder. After incubation of the dish at 37°C for 5 minutes, 100 μL culture medium was added. Cell suspension was aspirated and transferred a medium-filled 6-well plate. Cloning cells were maintained as same methods as the primary culture. Following experiments were done using this cloned immortalized cells (passage number > 15) and primary cells (passage number = 2).

### Immunoblotting analysis

Primary cells and BFCE-K4DT cells were homogenized in lysis buffer (1 M Tris-HCl at pH 7.4, 3 M NaCl, 1% Triton X-100, 6 mM sodium deoxycholate, and 0.5% protease inhibitor cocktail; Nacalai Tesque). The detailed method for western blotting was described in our previous report [15]. A rabbit polyclonal antibody against human cyclin D1 (1:5000, Medical & Biological Laboratories Co., LTD., Nagoya, Japan), a mouse monoclonal antibody against human CDK4 (1:2000, Medical & Biological Laboratories Co., LTD.), and an antibody againstá-tubulin (1:1000, Santa Cruz Biotechnology, Inc., Dallas, US) were used as the primary antibodies. The corresponding secondary antibodies (sheep anti-rabbit and anti-mouse IgG conjugated to horseradish peroxidase, 1:2000; GE Healthcare, Little Chalfont, Buckinghamshire, UK) were used for detection. Signals on the membranes were detected using an ECL kit (GE Healthcare), and the image was obtained with an ImageQuant LAS-4000 Mini system (GE Healthcare) under the condition (exposure time; 2 min, sensitivity; High).

### Detection of telomerase activity

Telomerase activity was detected with the telomere repeat amplification protocol (TRAP) method [16], using a TRAPeze Telomerase Detection Kit (Millipore, Billerica, US). Cell extracts were obtained from the established cells cultured on 6-well plates. Cell extract from 293 T cells was used as a positive control. The telomerase extension reaction was performed at 37°C for 90 min. PCR and polyacrylamide gel electrophoresis (PAGE) were conducted according to the manufacturer’s recommendations. To visualize DNA products, the gel was stained with GelRed^™^ (Biotium, Hayward, US), and the images were captured by using a UV transilluminator at 254 nm.

### Karyotype analysis of immortalized cells

Cells were treated with colcemid at a final concentration of 0.02 mg/ml on the day before harvesting in 15 passage numbers. After trypsinization, the cells were suspended in hypotonic solution and fixed in Carnoy’s fluid. Fixed cells were dropped onto a glass slide and stained using the G-band staining method. Fifty metaphase cells were analyzed for their karyotype. The results obtained from this analysis were displayed according to the International System for Human Cytogenetic Nomenclature (ISCN). This analysis was conducted in Nihon Gene Research Labolatories Inc. (Miyagi, Japan, test number: 15000611).

### Fluorescent immunohistochemistry for detection of cytokeratin and E-cadherin

An immunohistochemical study was performed on cells fixed with 4% paraformaldehyde in PBS for 20 min at room temperature on a 12-well plate. The fixed cells were washed with PBS, and incubated with 0.5% Triton X-100 in PBS for 30 min at room temperature for permeabilization. As a negative control for this staining, bovine fetal fibroblast cells (BFF NCC) were used [11]. After a wash with PBS, the cells were treated with 1% bovine serum albumin (SIGMA) in PBS as blocking buffer for 1 h at room temperature. Purified mouse anti-E-cadherin (BD Bioscience) and monoclonal antibody to cytokeratin 8 (EXBIO, Vestec, Czech Republic) were used as primary antibodies. The samples were exposed to primary antibody in blocking buffer at 4°C overnight. Cells were washed three times with PBS for 5 min and treated with Alexa Fluor 488-conjugated secondary antibody (Invitrogen) for 1 h at room temperature, while protecting from exposure to light. The nuclei were visualized by staining with 1 μg/ml of 4',6-diamidino-2-phenylindole (DAPI) (Dojindo Laboratories, Kumamoto, Japan). The stained images were detected by a fluorescence microscope system (FSX100, Olympus).

### Bacterial strains and culture conditions

*Salmonella enterica* serovar Enteritidis strain zSE1 isolated from Zambia [17] and Typhimurium wild type strain st1wt [18] bacteria were grown routinely in Trypcase Soy Broth (TSB) at 37°C for 18 h. Enterohemorrhagic *Escherichia coli* (EHEC) obtained by recloning of EDL931 (strain ATCC 35150) was grown routinely in Brain Heart Infusion (BHI) broth at 37°C for 20 h.

### Detection of EHEC adhesion

BFCE primary cells and BFCE K4DT cells were seeded in a 12-well plate to reach a density of 5.0 × 10^4^ cells/well and incubated for 48 hours at 37°C in an atmosphere containing 5% CO_2_. The medium was changed to DMEM with 10% FBS (without antibiotics) 2 hours before bacterial infection to eliminate any potential effects of the antibiotics, and cells were infected with bacteria at 3.0 × 10^6^ cells/well. To count the adhesive bacteria at 30, 60, and 120 minutes after the infection, wells were washed with PBS 3 times and disrupted with lysis buffer (PBS containing 0.1% (wt/vol) sodium dodecyl sulfate (SDS) (Wako Pure Chemical Industries, Ltd., Osaka, Japan) and 1% (vol/vol) Triton X-100). Lysate containing EHEC was 10-fold diluted with PBS and plated on nutrient agar for determination of adhesion bacterial counts.

### Gentamicin Protection Assay

Conditions of cell seeding and infection of *Salmonella* were same as the methods of EHEC adhesion assay. To count the total (adhesive and invasive) bacteria at 30, 60, and 120 minutes after the infection, wells were washed with PBS 3 times and disrupted with lysis buffer. To the count for the invasive bacteria, cells were washed with PBS 3 times and incubated with 100 μg/mL gentamicin-containing fresh culture medium for 2 hours post-infection, which results in adhesive bacteria were killed. Gentamicin treated cells were lysed with the lysis buffer after PBS wash 3 times and harvested. Each lysis buffer containing bacteria were 10-fold diluted with PBS and plated on nutrient agar for determination of total and invasive bacterial counts.

### Fluorescent immunohistochemistry for detection of Bacterial adhesion and invasion

For the assay, BFCE-K4DT cells were seeded on 24-well tissue culture plates at a density of 1 × 10^5^ cells/well and incubated overnight. Before infection, cells were incubated for 2 h in a medium without antibiotics. They were then infected for 120 min at 37°C with a bacterial suspension at 5.0 × 10^5^ cells/well. Cells were washed in PBS to remove non-adherent bacteria. After 1 h blocking with 1% BSA in PBS at room temperature, extracellular bacteria were stained with an anti-*Salmonella* serotype-specific antibody (*S*. Typhimurium, O9 serum and *S*. Enteritidis, O4 serum; diluted 1:300, Denka Seiken Co., LTD., Tokyo, Japan), which was followed by incubation with Alexa 594-labelled goat anti-rabbit antibody (Life Technologies, diluted 1:200) for 1 h at room temperature. Cells were permeabilized for 5 min with 0.2% Triton X-100, and total bacterial counts were determined by staining with antiserum against *S*. Typhimurium, O9 serum, or antiserum against *S*. Enteritidis, O4 serum (diluted 1:300, Denka Seiken Co., LTD.), followed by incubation with Alexa 488-labelled goat anti-rabbit antibody (Life Technologies, diluted 1:200) for 1 h at room temperature. In this way, internalized bacteria (green) were distinguished from extracellular bacteria (yellow, due to an overlay of red and green fluorescence). The nuclei were visualized by staining with 1 μg/ml of DAPI, and the staining patterns were detected by a fluorescence microscope (FSX100, Olympus, Tokyo, Japan).

### Scanning Electron microscopy (SEM) analysis

BFCE cells were seeded on the 8 well Millicell^®^ EZ SLIDE (Millipore) at 5.0 ×10^4^ cells. Cells were incubation overnight to detect bacterial adhesion. For the detection of bacterial adhesion, cells were changed media without antibiotics 2 h before the bacterial infection. They were then infected for 120 min at 37°C with a bacterial suspension at 3.0 ×10^6^. To prepare the SEM observation, cells were washed PBS and fixed with 2% glutaraldehyde in 0.1 M cacodylate buffer for 2 h at 4°C and dehydrated by 70%, 90% and 100% ethanol for 15 min respectively. Fixed and dehydrated cells were coated with platinum-palladium and observed by SEM (S4200, Hitachi, Tokyo, Japan).

### Detection of Toll-like receptors (TLRs) transcription

BFCE-K4DT cells were seeded at 3.0 ×10^5^ cells to a 6 well plate and incubated for 48 hr. Total RNA was isolated by using NucleSpin RNA Plus and Reverse transcription was performed by using PrimeScript^™^ RT Master Mix (TaKaRa Bio Inc., Shiga, Japan) according to manufacturer’s instructions. Realtime PCR was done on a Thermal Cycler Dice Single (Takara) using SYBR Premix Ex Taq (TaKaRa). Quantitative PCR was performed with appropriate primers (S1 Table) [19] under the following conditions: 30 sec holding at 95°C, 40 cycles of 2 step PCR at 95°C for 5 sec and 60°C for 30 sec, Dissociation curve at 95°C for 15 sec, 60°C for 30 sec and 95°C for 15 sec.

### Statistical analysis

To test for statistical differences in this research, we used Student’s *t* test. A *P*-value less than 0.05 was considered statistically significant. Data were expressed as mean ± Standard Deviation (SD).

## Results

### Immortalization of bovine fetal colon epithelial (BFCE) cells and their division rate

**Fig 1:**
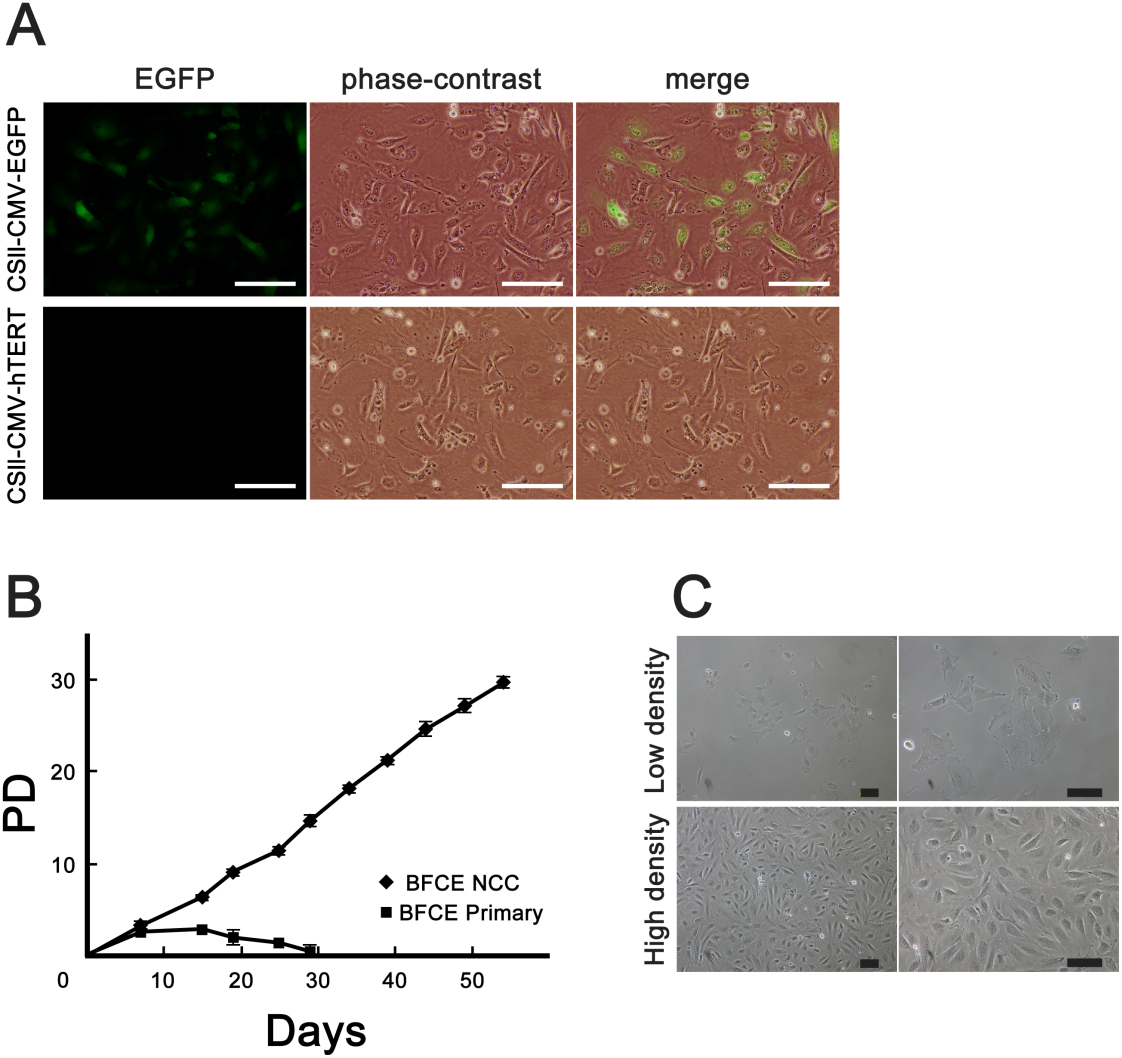
The efficiency of virus infection, cell morphology, and population doubling of the established BFCE-K4DT cells. (A) Green fluorescence was detectable in primary cells transfected with CSII-CMV-EGFP vector (upper panels), while cells transfected with CSII-CMV-TERT vector were not fluoresce (lower panels). Scale bars: 50 μm. (B) The results of the population doubling assay in BFCE primary cells (squares) and BFCE-K4DT cells (diamonds) were plotted. Experiments were carried out in triplicate, and the averages and standard deviations (SD) were calculated. (C) The morphologies of the established cell line after cloning (after 15 times passage). BFCE-K4DT cells show an epithelial-like rounded shape in low-density culture (upper panels) and sheet-like morphology in high-density culture (lower panels). Scale bars: 100 μm.

To monitor the efficiency of recombinant virus infection, we exposed primary bovine fetal colon cells to CSll-CMV-EGFP as well as simultaneously introduction of CSll-CMV-TERT, -cyclin D1, and -hCDK4R24C. Primary bovine colon cells were efficiently infected with the EGFP-expressing virus, resulting in a high percentage of green fluorescence-positive cells (Fig 1A). We named the BFCE cells transduced with mutant CDK4, Cyclin D1, and TERT as “BFCE-K4DT” (Bovine Fetal Colon Epithelial cell established with CDK4, cyclin D1 and TERT). To determine if the BFCE-K4DT cells were fully immortalized, we evaluated the cell proliferation rate by measuring the population doubling (PD) value. Fig 1B shows the PD value of BFCE primary cells (diamond) and BFCE-K4DT cells expressing CDK4R24C, cyclin D1, TERT (square). BFCE-K4DT cells proliferated more rapidly than did BFCE primary cells, and BFCE primary cells arrested cell proliferation at the 5th passage.

BFCE-K4DT cells showed epithelial cell-like morphologies, in both low and high cell density conditions (Fig 1C). When we compared the cell morphology of BFCE-K4DT cells with that of the original BFCE, there was no significant difference between them (data shot shown).

### Detection of CDK4R24C and cyclin D1 expression and telomerase activity

**Fig 2:**
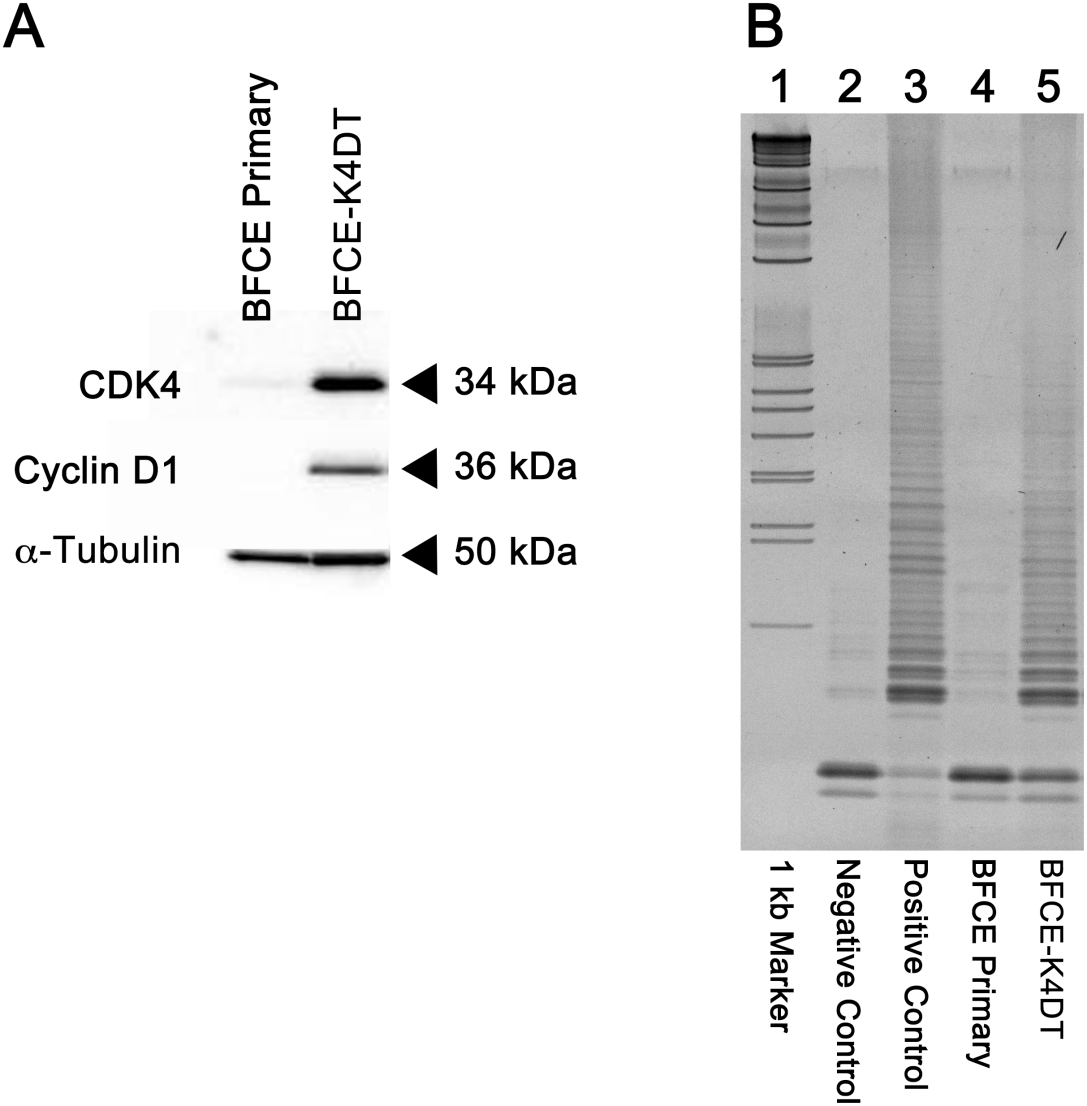
Results of the immunoblotting analysis and TRAP assay. (A) Detection of CDK4 and cyclin D1 by immunoblotting. Lane 1, BFCE primary cells; Lanes 2 and 3, BFCE-K4DT cells. (B) Detection of telomerase activity by TRAP assay. Lane 1, 1 kb ladder marker; lane 2, CHAPS buffer as a negative control; lane 3, 293T cells as a positive control; lane 4, BFCE primary cells; lane 5 and 6, BFCE-K4DT cells. The 6-bp ladders were detectable in lanes of positive control and BFCE-K4DT cells. Each experiment was performed in duplicates after cloning (primary cells: 2 times passage, K4DT cells: 15 times passage) and representative data were shown here.

The expression of mutant CDK4 and cyclin D1 proteins was detected using western blotting, and the results are shown in Fig 2A. As expected, primary BFCE cells did not show any signals for these proteins (Fig 2A, lane 1), whereas BFCE- K4DT cells showed high protein expression for both CDK4 and Cyclin D1 proteins (Fig 2A, lane 2).

We next investigated telomerase activity with the TRAP assay [18]. We observed 6-bp ladders in 293T cells (used as positive control) and minimal activity levels in the negative control, verifying the reliability of the detection method. BFCE primary cells were negative for the activity (Fig 2B, lane 3), but both BFCE-K4DT 1 and 2 showed high levels of telomerase activity (Fig 2B, lanes 5), indicating that the introduced TERT was properly expressed in BFCE-K4DT cells.

### Karyotype analysis inimmortalized cell line

**Fig 3:**
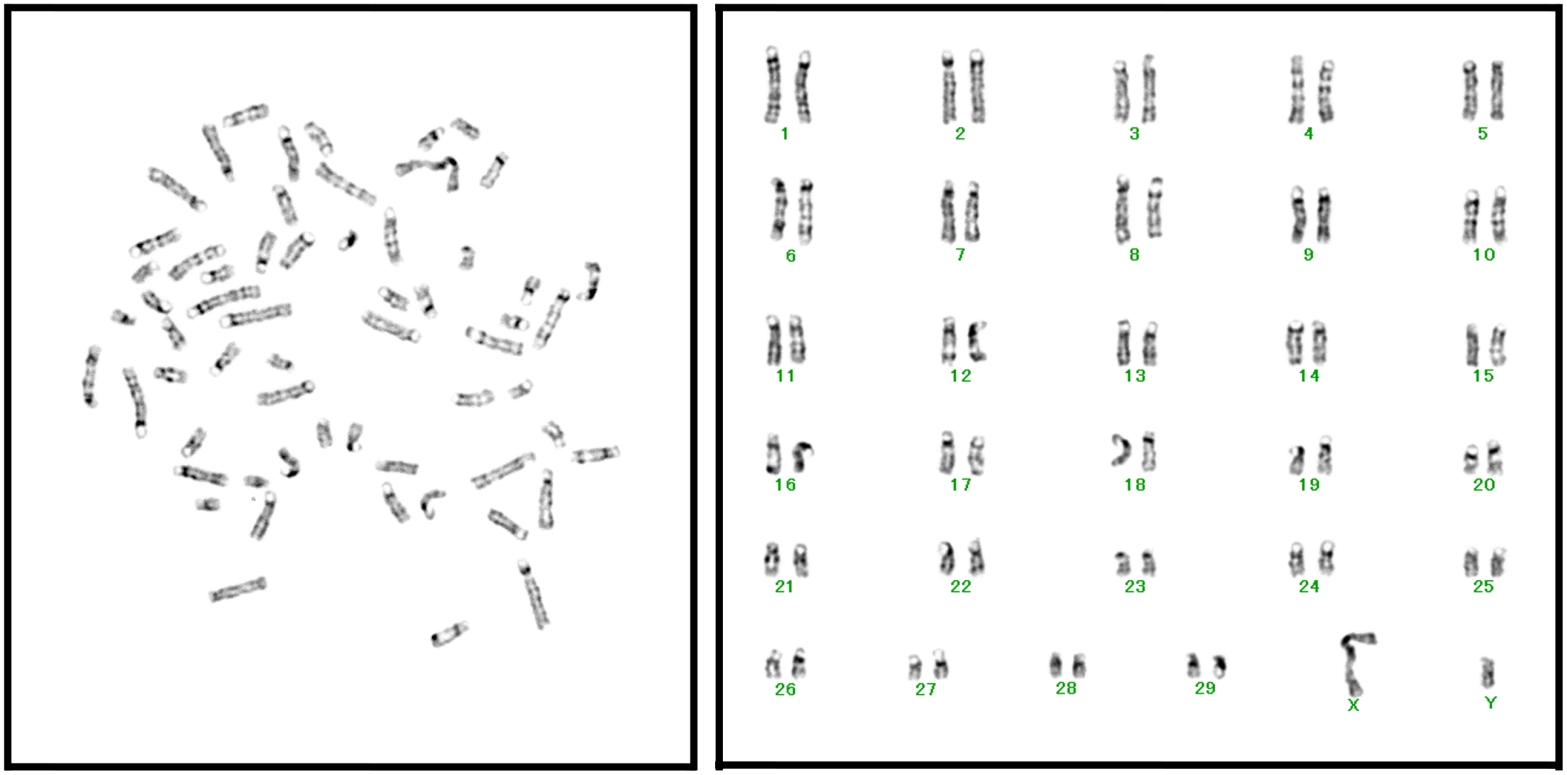
Karyotype analysis of established BFCE-K4DT cells. All mitotic chromosome spreads (50 of 50 examined) from BFCE-K4DT cells showed a 2n = 60XY pattern. This analysis was performed by using cloned cells after 15 times passage.

Chromosomal analysis demonstrated that the established BFCE cells exhibited a normal 60XY diploid karyotype (Fig 3). This indicated that introduction of the human cyclin D1, CDK4R24C, and TERT expression constructs immortalized the colon epithelial cells with an intact karyotype.

### Detection of epithelial markers in established BFCE-K4DT cells

**Fig 4:**
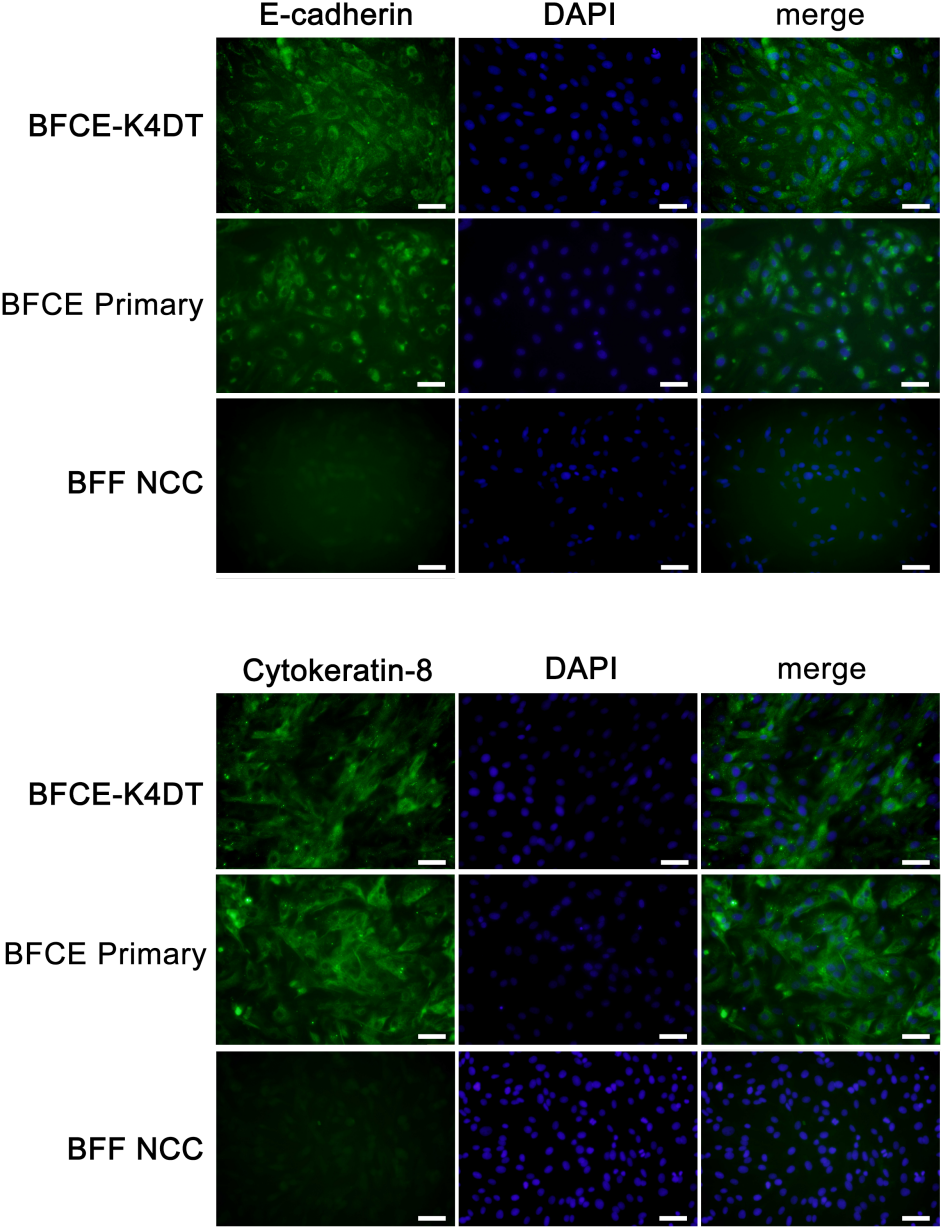
Fluorescent immunohistochemical staining for detection of epithelial markers. BFCE-K4DT cells (passage number = 15) and BFCE-Primary cells (passage number = 2) were positive for E-cadherin and Cytokeratin-8, whereas BFF NCC cells (passage number was unknown) were negative for both markers. Scale bars: 50 μm. Each staining was carried for 3 times and representative pictures were shown here.

E-cadherin and cytokeratin are representative markers expressed in epithelial cells. To confirm that the established BFCE-K4DT cell line had epithelial features, we conducted immunohistochemical analysis of BFCE-Primary cells, BFCE-K4DT cells and bovine fetal fibroblast cells (BFF NCC) that were established by using same methods. Both epithelial markers were detected in BFCE-Primary cells and BFCE-K4DT cells (Fig 4). Interestingly, these epithelial markers expression was detected throughout the cytoplasm in BFCE-K4DT cells. On the other hand, minimal signals were detected in BFF NCC cells (Fig 4). These findings indicate that BFCE-K4DT cells are derived from epithelial cells.

### Adhesion and invasion analysis using *Salmonella* spp.

**Fig 5:**
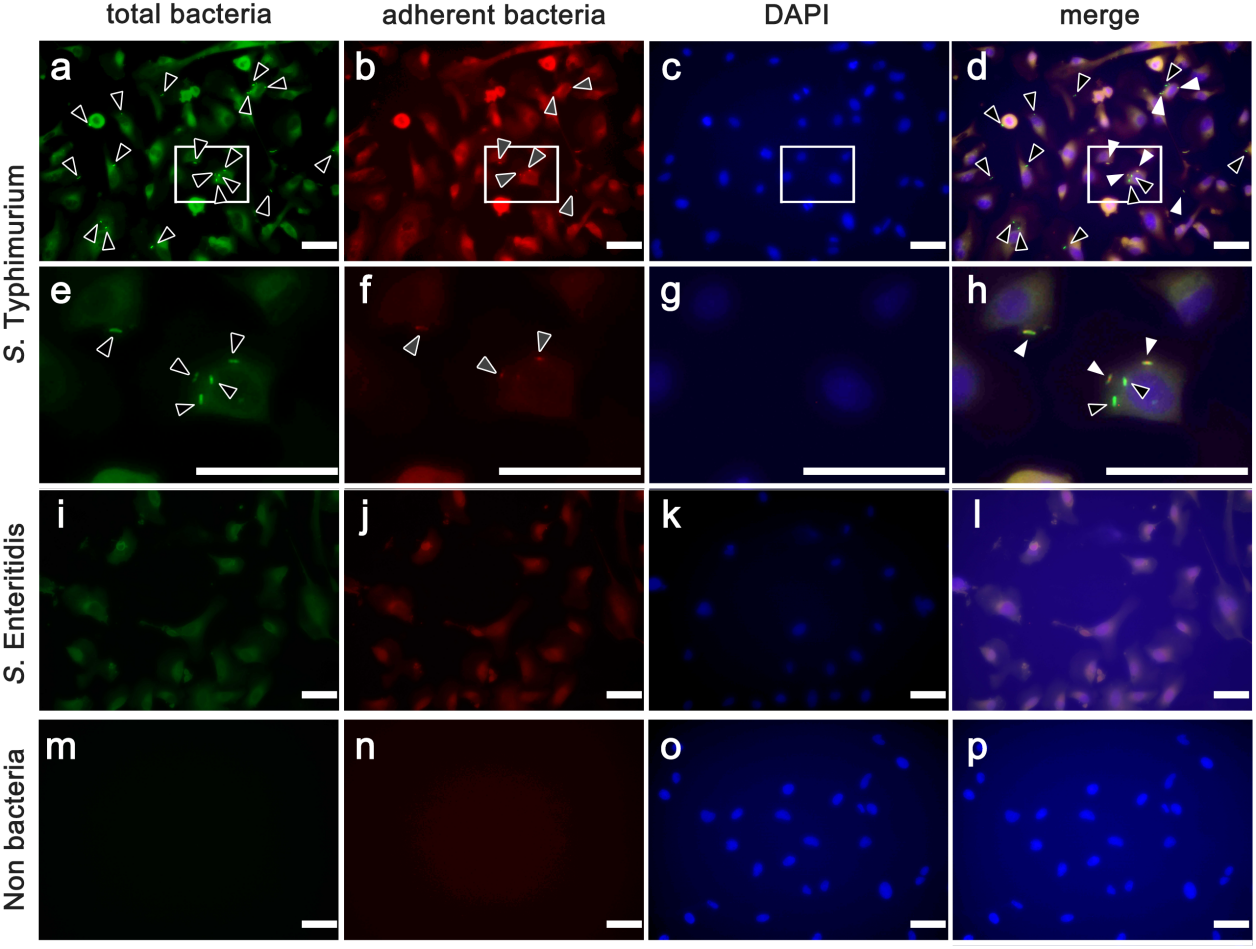
Fluorescence microscopic images of cells subjected to the adhesion and invasion assay. *S*. Typhimurium (a-d: low-power fields and e-h; high-power fields), *S*. Enteritidis (i-l) after infection, and non-infected control (m-p)) in BFCE-K4DT cells (passage number = 15). Total bacteria were stained green (a, e, i, and m; black arrows), adherent bacteria were stained red (b, f, j, n; white arrows), nuclei of cells were stained blue, and merge images of extracellular bacteria (stars) and intracellular bacteria (black arrows) appear yellow and green respectively (d, h, l, p). Each staining was carried for 3 times and representative pictures were shown here.

With this staining assay, *S*. Typhimurium showed positive signals with Alexa 488 (Fig 5a and 5e, black arrowheads) and Alexa 594 (Fig 5b and 5f, white arrow heads). In merged images, adherent *S*. Typhimurium yielded a yellow fluorescence pattern (Fig 5d and 5h, stars) and intracellular *S*. Typhimurium was revealed by green fluorescence (Fig 5, 5d and 5h, black arrows). These results indicate that *S*. Typhimurium has affinity for the surface cellular membrane and can invade the established BFCE-K4DT cells. However, BFCE-K4DT cells exposed to *S*. Enteritidis, as well as non-infected BFCE-K4DT cells, did not show any intense dots of staining in the cytoplasm (Fig 5, 5i–5l and 5m–5p).

### Quantification and observation of bacterial adhesion and invasion

**Fig 6:**
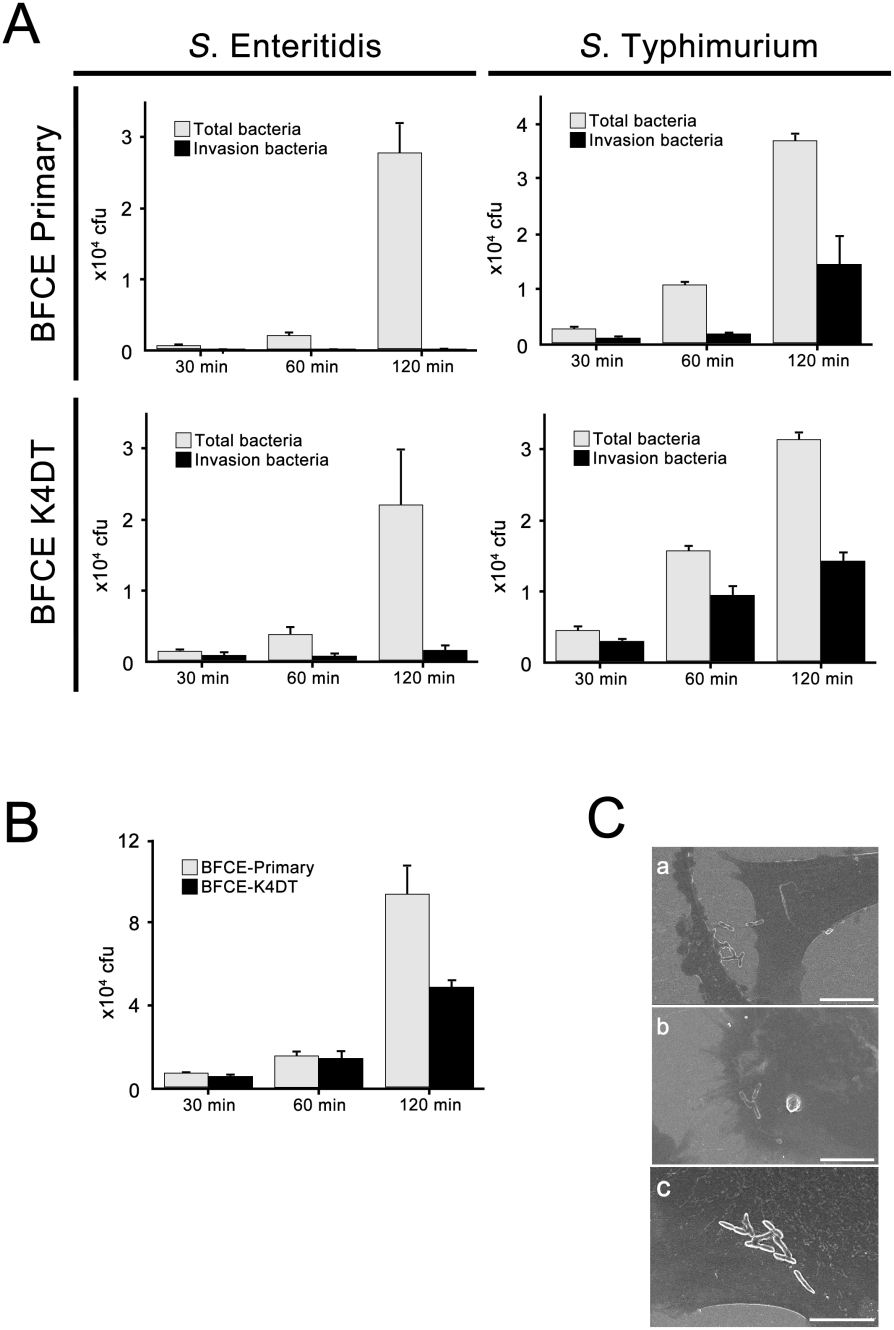
Quantification of bacterial adhesion and invasion and SEM microscopy. (A) Total *S*. Enteritidis or *S*. Typhimurium were harvested after each infection time (30, 60, 120 min) and plated in agar dishes to count the colony number (gray bars). Invasive bacteria were also harvested after 2 h gentamycin treatment at the each time point and count the colony by same procedures as total bacteria (black bars). (B) EHEC adhesion to BFCE-Primary cells (gray bars) and BFCE-K4DT cells (black bars) were also quantification by same methods as *Salmonella* infection. (C) SEM observations revealed the adhesion of *S*. Enteritidis (a), *S*. Typhimurium (b) and EHEC (c) to established BECE-K4DT cells (bars: 10 μm). Experiments were carried out in triplicate, and the averages and standard deviations (SD) were calculated (A and B) and representative figures were shown (C).

Fig 6A is showing the results of gentamycin protection assay using *S*. Enteritidis and *S*. Typhimurium. In BFCE-Primary cells, *S*. Enteritidis was detectable as total bacteria in time-dependency but not as invasion bacteria, which indicates that *S*. Enteritidis hardly invade BFCE-Primary cells while can adhere the cell surface. Although this tendency was observed in BFCE-K4DT cells, invasive *S*. Enteritidis was able to detect even in 30 min after infection. *S*. Typhimurium could adhere both BFCE-Primary and–K4DT cells as same as Primary cells, and also invade in time-dependency more than *S*. Enteritidis. Results of fluoroscopy assay described above and these quantifications suggest that established BFCE-K4DT cells have host specificity originated from primary cells, and immortalized BFCE-K4DT cells can be more susceptible for invasion of salmonella than primary cells.

We sought to identify the adhesion of other bacterium, EHEC, and show the result in Fig 6B. The number of adhesion EHEC in both Primary and K4DT cells increased time-dependent manner. At 120 min after infection, adhesion of EHEC on BFCE-Primary was more than -K4DT cells. This bias could also be confirmed in Salmonella infection (Fig 6A), however observed more prominently in EHEC infection.

Scanning Electron Microscopy produced detailed images that show bacterial adhesion on the cell surface. *S*. Enteritidis (Fig 6Ca) and *S*. Typhimurium (Fig 6Cb) tended to attach the cell edge while EHEC was adhering cell surface (Fig 6Cc).

### Expression of TLRs in established cells

Quantitative RTPCR revealed that established BFCE-K4DT cells transcribed TLR 1, TLR 2 and TLR 3, but TLR4, 5 and 6 were could not detectable. This result was different from previous study that primary colonocyte cells expressed TLR 1, TLR 3, TLR 4 and TLR 6 [19] (Table 1).

**Table 1:** Toll-like receptors (TLRs) gene expression in established BFCE-K4DT cells.

TLR genes transcription	TLR 1	TLR 2	TLR3	TLR4	TLR5	TLR6	Reference
This study	+	+	+	-	-	-	
Bridger *et al*.	+	-	+	+	-	+	[19]

+: positive,

-: negative

## Discussion

Intestinal epithelial cells are exposed to serious risk of the pathogenic microbes although have an important role for the uptake of nutrients and fluids. Previous studies have attempted to establish intestinal epithelial cell lines from various animals, such as cat [20], mouse [21] and cattle [22, 23] by the introduction of Simian Vacuolating Virus 40 large T antigen. Transfection of this gene is the most frequently used to immortalization not only intestinal cells but also various cells. These established cell lines, however, include the risk for the change of the original nature of the primary cells [7, 8].

For the establishment of a cell line, the first difficulty to overcome is the Hayflick limit [24]. Primary cells cannot proliferate indefinitely due to cellular senescence. As a solution to this limitation, we introduced human Cyclin D1, mutant CDK4, and TERT. The version of CDK4 we transfected has a mutation (R24C) at the binding site for the senescence protein p16. When a normal cell is exposed to cellular stress, or reaches the senescence point, p16 protein accumulates in the cells [25, 26]. The p16 protein binds to CDK4 and negatively regulates the activity of CDK4-Cyclin D1 complex. However, p16 cannot suppress the CDK4R24C-Cyclin D1 complex. Due to the constitutive activity of the CDK4R24C-Cyclin D1 complex, phosphorylation and inactivation of retinoblastoma protein (pRB) is maintained, resulting in an accelerated rate of cell growth. This method of cell immortalization was initially reported in humans [9], and we previously reported that this immortalization method could be applied to a wide variety of animals, since CDK4 and Cyclin D1 are evolutionally conserved [11, 12]. In the present study, we established a BFCE-K4DT cell line, derived from bovine fetal colon epithelial cells via transfection of three genes, human Cyclin D1, mutant CDK4, and telomerase. This cell line showed an accelerated speed of cell proliferation and kept on proliferating after stopping its growth in BFCE-Primary cells (passage number = 5) as a result of the expression of these three genes. Furthermore, karyotype analysis revealed that established BFCE-K4DT cells maintain an intact karyotype after passage number 15. From these results, we succeeded in establish immortalized cell line from primary cells having comparatively weak proliferative capacities.

The BFCE-K4DT cells showed positive expression of cytokeratin 8 and E-cadherin, which are markers of epithelium-derived cells although these expressions were not strong in the cell-to-cell contact region. Thus, tight junction of BFCE-K4DT cells is not strong even when cells are confluence. Furthermore, BFCE-primary cells were also not strong expression levels of these epithelial markers in the cell-to-cell contact region, which suggests that this feature was not caused by the method in this study but the native character. Furthermore, SEM analysis in this study revealed surface of BFCE-K4DT cells (Fig 6C), and we could not recognized rough and slender microvilli-like structure observed in other research reported [22] even when we maintained BFCE-K4DT cells for 1 week after confluency in the culture (data not shown), but microvilli-like projections were detectable (Fig 6Cc). It was known one of the reasons why a loss of the epithelial characteristics including cell-to-cell junction is caused by epithelial mesenchymal transition (EMT) [27] resulting from cell disaggregation during the period of epithelial cell culture condition [28]. Hanako *et al*. have reported that established bovine endometrial epithelial cells by transfecting HPV E6, E7 and human TERT genes showed positive staining both of cytokeratin and vimentin, stromal marker [29]. We sought to establish the new cell line because the expression of these oncogenic proteins could lead to change the original nature of the primary cells. These observations suggested that further challenges are necessary to understand the flexible and dynamic cell environments by using various types of cell lines, and established BFCE-K4DT cells could be a valuable tool.

Salmonellosis is an important disease of cattle, most often caused by *S*. Dublin and *S*. Typhimurium. *S*. Typhimurium is frequently detected in calves around 2 months of age, in which it induces diarrhea [30–32] and also infect a wide range of domesticated or wild animals, as well as humans [33]. *S*. Enteritidis is most often isolated from chickens and eggs [34], and is also one of the major causes of human food-borne gastroenteritis worldwide [35]. Interestingly, many *S*. Typhimurium were detected as intense dots of positive staining when added to BFCE-K4DT cells, which was explained as the presence of the bacterium in the cytoplasm of the cells. However, *S*. Enteritidis did not show any intense positive signals in the cytoplasm, indicating a difference in the ability of *S*. Typhimurium and *S*. Enteritidis to invade bovine colon epithelium. Furthermore, Gentamycin protection assay revealed that the numbers of invasive S. Enteritidis were smaller than that of *S*. Typhimurium in support of immunofluorescent staining despite higher dose of Salmonella than Fluorescent immunohistochemistry. Previous study revealed that there are differential immune responses to S. Typhimulium, *S*. Dublin, and *S*. Enteritidis in bovine peripheral blood leukocytes [36]. These suggests that this detected difference of invasion number between *S*. Typhimurium and *S*. Enteritidis might account for their distinct host infection associations and can be a possible factor of immune response differentiation.

Toll-like Receptors (TLRs) contribute to host resistance to microbial pathogens, and each TLR bind targets as microbial ligands, such as tri-acylated lipopeptide (TLR 1), lipoprotein (TLR 2), double strand RNA (TLR 3), lipopolysaccharide (LPS) (TLR 4), flagellin (TLR 5) and Di-acyl lipopeptide (TLR 6) [37, 38]. In this research, we could detect the TLR 1, TLR 2 and TLR 3, but not TLR 4, TLR 5 and TLR 6. On the other hand, Bridger *et al*. have reported that bovine primary colonic cells established by them harbored mRNA specific for TLR 1, TLR 3, TLR 4 and TLR 6 [19]. These results show there are differences of expressing TLR genes among bovine colonic cells, and new bovine colon cell line establishment will lead to an opportunity for deeper understanding of the interactions between pathogenic bacteria and bovine host. TLR 2 mediates cellular responses to various infectious pathogens and their products including yeast cell walls, whole mycobacteria, peptidoglycan [39–44]. TLR 2 activities for recognition of these ligands, however, are known to complex other TLRs, such as TLR 1 and TLR 6 [45]. Q-RTPCR showed TLR 1 and TLR 2 were transcribed in BFCE-K4DT cells, so that this cell line is a useful model for investigation of the interaction between infectious pathogens and TLR 1/2 heterodimer complex. Although established BFCE-K4DT cell did not show gene expression TLR 6, they could be useful in vitro model for discussing about interaction with TLR 2 and the downstream signaling of TLR 2/6 heterodimer complex. Tabeta et al. have reported that TLR3 deficient mice are hyper-susceptible to mouse cytomegalovirus infection, which indicates TLR3 has a protective role against viral infection [45]. TLR3 is expressed in cell endosomal compartments can recognize viral nucleic acids [46]. Established BFCE-K4DT cells are expressing TLR3, thus this cells are suitable for the study accompanied with not only bacterial infection but also viral infection. In this study, TLR 4 playing a key role in the recognition of LPS and TLR 5 that is triggering cardiac innate immune responses and causing acute contractile dysfunction when combined with its ligand flagellin [47] were not detectable. Thus, established cells can be considered pathways via these receptors’ ligand were inactivated, such as MyD88- and TIRAP- dependent pathways [48]. TLRs-deficient mice were developed to examine the role of TLRs *in vivo* [49–54]. BFCE-K4DT cells can be suited for *in vitro* monitoring the effects of these ligands without these TLRs and them downstream pathways as well as TLR 1, 2, 3—mediated reaction confirmed in BFCE-K4DT.

These results need to be further evaluated with various types of bacterial strains, and our established immortalized colon epithelium-derived cell line having many distinctive features will continue to be a useful tool to investigate the molecular mechanisms underlying intestinal bacterial and viral infection.

## Supporting Information

S1 Table Sequences of primers for detection of TLRs gene expression. Primers were designed according to previous report [19].
(DOCX)
