# Supplementary material for: Immortalization of Fetal Bovine Colon Epithelial Cells by Expression of Human Cyclin D1, Mutant Cyclin Dependent Kinase 4, and Telomerase Reverse Transcriptase: An In Vitro Model for Bacterial Infection
Source: PLoS One. 2015 Dec 1;10(12):e0143473. doi: 10.1371/journal.pone.0143473 (PMC4666463; doi:10.1371/journal.pone.0143473)
Supplement: S1 Table — Primers were designed according to previous report [19]. (DOCX) [file pone.0143473.s001.docx]

**S1 Table Sequences of primers for detection of TLRs gene expression.**

| Primer name | Forward (5’ - 3’) | Reverse (5’ - 3’) |
| --- | --- | --- |
| TLR1 | TTCCAGAGCTGCCAGAAGAT | GAGATTGTGGTGGGCAAAGT |
| TLR2 | CAGCAACTGAAGACGTTGGA | CACCACTCGCTCTTCACAAA |
| TLR3 | CCCCAGTCTCACAGAGAAGC | CCTGTGAGTTCTTGCCCAAT |
| TLR4 | TGCTGGCTGCAAAAAGTATG | TCTGCAGGACGATGAAGATG |
| TLR5 | TGCATCCAGATGCTTTTCAG | CCTTCAGCTCCTGGAGTGTC |
| TLR6 | AGGCCAAGTATCCAGTGACG | GAGATTGTGGTGGGCAAAGT |
| b-actin | CCAGACAGCACTGTGTTGGC | GAGGAAGCTGTGCTACGTCGC |

Primers were designed according to previous report [19].
